# Supplementary material for: The interactome of intact mitochondria by cross-linking mass spectrometry provides evidence for coexisting respiratory supercomplexes
Source: Mol Cell Proteomics. 2017 Dec 8;17(2):216–32. doi: 10.1074/mcp.RA117.000470 (PMC5795388; doi:10.1074/mcp.RA117.000470)
Supplement: Supplemental Data [file supp_RA117.000470_133922_0_supp_23978_fzffwf.zip › spectra_annotation/mito_spectra_annotation/54-1-1-1-4-1.pdf]

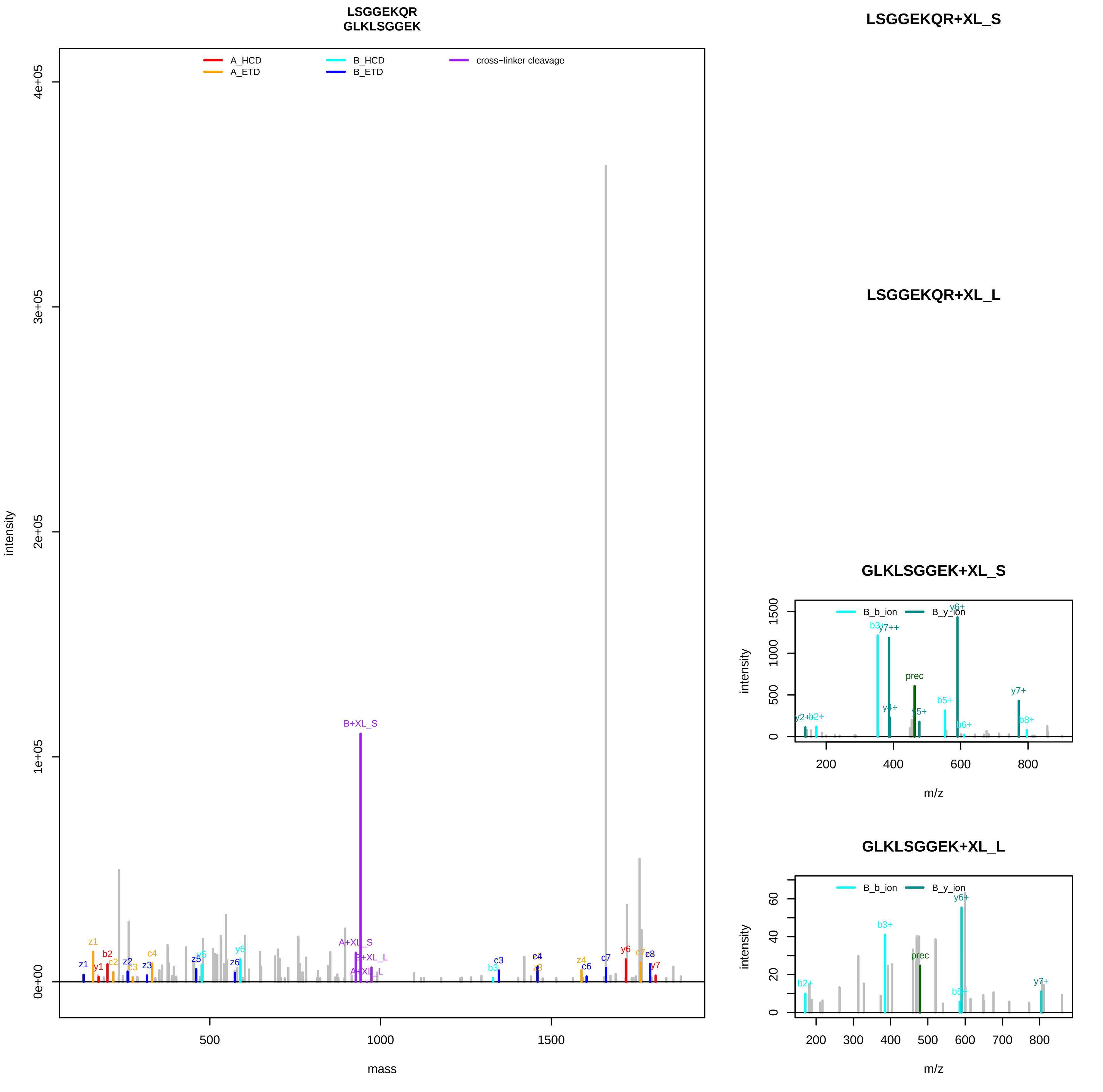
LSGGEKQR
GLKLSGGEK
A_HCD
A_ETD
B_HCD
B_ETD
cross−linker cleavage
intensity
mass
4e+05
3e+05
2e+05
1e+05
0e+00
500
1000
1500
B+XL_S
A+XL_S
B+XL_L
A+XL_L
z1
y1
b2
c2
z2
c3
z3
c4
z5
y5
z6
y6
b3
c3
c4
z3
z4
c6
c7
y6
c7
c8
y7
LSGGEKQR+XL_S
LSGGEKQR+XL_L
GLKLSGGEK+XL_S
B_b_ion
B_y_ion
y6+
b3+
y7++
prec
y4+
y5+
b5+
b6+
y7+
b8+
y2+
b2+
m/z
1500
1000
500
0
200
400
600
800
GLKLSGGEK+XL_L
60
40
20
0
300
500
700
